# Black Coloured Urine following Organophosphorus Poisoning: Report of Two Cases

**DOI:** 10.1155/2014/706021

**Published:** 2014-03-04

**Authors:** Aneesh Basheer, Sudhagar Mookkappan, Vijay Shanmugham, Nagarajan Natarajan, Kiran Kulirankal

**Affiliations:** Department of General Medicine, Pondicherry Institute of Medical Sciences, Pondicherry 605014, India

## Abstract

Organophosphorus poisoning is common in rural Asia. Clinical features result from overactivity of acetylcholine receptors. Blackish discoloration of urine is not a feature of organophosphorus poisoning. Only one case of black colored urine following quinalphos poisoning has been reported in literature. We report two cases of organophosphorus poisoning from two different compounds, following which patients passed black colored urine, in the absence of haemolysis or rhabdomyolysis. These cases indicate that blackish discoloration of urine in organophosphorus poisoning might not be as uncommon as it was believed to be. Besides, urinary excretion of metabolites might be an underlying mechanism, rather than hemolysis.

## 1. Introduction

The public health importance of organophosphorus poisoning is reflected in the huge number of deaths due to suicidal and accidental toxicity from these compounds [1]. History of exposure, characteristic signs and symptoms of toxicity, and low serum cholinesterase levels make the diagnosis almost certain [2]. Intermediate syndrome causing respiratory failure is one of the most dreaded complications [3]. Although atypical complications like pancreatitis and haemolysis have been described [4, 5], only one case of blackish discoloration of urine has been reported in literature, following quinalphos poisoning [6]. This report describes two cases sharing this atypical manifestation, but due to different compounds.

## 2. Case Report

**Figure 1:**
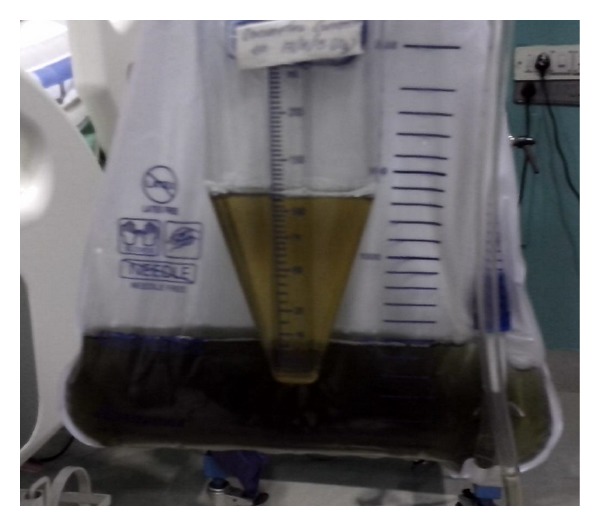
Black urine 9 hours after consumption of monocrotophos in the first patient.

The first patient was a 26-year-old male farmer, brought to the emergency room 3 hours after intentional ingestion of 100 mL of monocrotophos (organophosphorus compound). He was conscious and oriented with a heart rate of 56/minute and blood pressure of 110/60 mm Hg. His pupils were pinpointed and he had fasciculations. Examination of chest revealed bibasal fine crackles. Gastric lavage was administered and he was started on infusion of atropine sulfate titrated according to clinical response. In view of diaphragmatic weakness, he was intubated and ventilated. His investigations showed haemoglobin of 13.5 g% with normal leucocyte and platelet counts. Renal and liver functions were normal. Serum cholinesterase levels were low (650 U/L; normal: 3500–8500 U/L). After 6 hours of hospitalization, he started passing black colored urine (Figure 1). We evaluated this uncommon manifestation and found no evidence of haemoglobin or myoglobin in the urine. Besides, creatine phosphokinase levels were found to be normal, ruling out the possibility of rhabdomyolysis. The patient developed ventilator associated pneumonia on the 3rd day. Endotracheal aspirate culture showed MRSA, which responded to clindamycin. Patient was weaned off ventilatory support on day 6. Discoloration of urine resolved over a period of 8 days and patient was discharged with no further complications.

**Figure 2:**
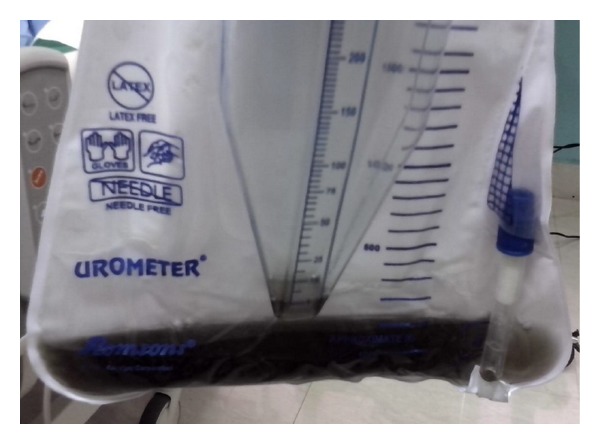
Black urine on day 2 of ingestion of fenthion in the second patient.

The second patient, a 39-year-old male diabetic, was admitted to the intensive care unit following consumption of 25 mL of ethion, 8 hours prior to hospitalization. He had already received gastric lavage and 10 mg of atropine from a primary care hospital. On examination, he had a heart rate of 98/min and blood pressure of 120/70 mm Hg with 4 × 4 mm pupils bilaterally reacting to light. His chest was clear and neurologic examination was normal. Over the next 3 hours he developed weakness of the neck flexors and respiratory muscle weakness. He was mechanically ventilated and started on atropine. His baseline investigations were normal except for hyperglycemia and a low serum cholinesterase (970 U/L). On the 2nd day, his urine showed blackish discoloration without any reduction in hourly output (Figure 2). In this patient too, workup for intravascular hemolysis and rhabdomyolysis turned negative. The patient was successfully weaned off the ventilator by day 7 and his urine became clear over the next 24 hours.

## 3. Discussion

Organophosphorus poisoning is common in rural Asia, accounting for 40% of the estimated 500,000 suicide related deaths annually [7, 8]. Case fatality rate is 15–30% in these rural areas [7]. These compounds are easily accessible and marketed in several strengths and combinations. They inhibit several members of the esterase group of enzymes, especially acetylcholinesterase and butyrylcholinesterase [2]. The former is predominantly found in the synaptic clefts and on red-cell membranes, while the latter is seen in the plasma (plasma cholinesterase or pseudocholinesterase) [2]. Metabolism of organophosphorus compounds in humans is mostly achieved through hepatic detoxification and to a lesser extent by extrahepatic pathways involving cytochrome P450 and flavin-containing monooxygenases [9]. The major metabolites of monocrotophos excreted in urine are N-methyl acetoacetamide and 3-hydroxy-N-methyl butyramide.

Though most of the organophosphorus pesticides are more potent inhibitors of butyrylcholinesterase, the well described clinical manifestations of organophosphorus poisoning are the result of acetylcholinesterase inhibition. Excessive accumulation of acetylcholine and subsequent hyperstimulation of acetylcholine receptors in the autonomic nervous system, central nervous system, and neuromuscular junctions appear to be the underlying mechanism of toxicity [2]. Clinically apparent effects have not been attributed to inhibition of butyrylcholinesterase.

The common clinical features of organophosphorus toxicity are summarized in Table 1 [10–12]. While all the features may not be present in any one individual, bradycardia, coma, and respiratory failure from intermediate syndrome are potentially fatal manifestations. Apart from excessive urination, abnormalities of urine color have not been part of traditional teaching as well as literature on organophosphorus poisoning. To date only one case of black urine has been described following organophosphorus poisoning [6], where the patient succumbed. The report highlighted the rare instance of urinary discoloration with regard to a single compound, quinalphos [6]. We report the occurrence of a similar phenomenon in two patients, who had ingested two different organophosphorus compounds. As a logical conclusion, it appears to be a class effect of organophosphorus compounds or their metabolites rather than an isolated compound-specific manifestation.

Another significant observation is the absence of any evidence for hemolysis in our patients. High coloured urine, ranging from black to red, is described in a number of conditions like dehydration, obstructive jaundice, alkaptonuria, and ingestion of several drugs like rifampicin, chloroquine, and iron sorbitol as well as toxins like cresol, copper, and phenol [13]. Among many other causes hemolysis and rhabdomyolysis require special attention [13]. Both these conditions signify serious underlying pathology and may lead to life threatening complications including acute renal shutdown. In the case reported by Viswanathan [6], the proposed mechanism for blackish discoloration of urine was hemolysis caused by quinalphos. This was evidenced by the presence of haemoglobin in urine, the disappearance of which correlated with the clearing of urine. Haemolysis attributable to specific organophosphorus compounds has been reported in literature [5], though urine color has not found place in these reports. Thus, the plausible explanation for black urine in our patients is the excretion of some common metabolite(s) of organophosphorus compounds. The overlap between time to clinical recovery and time for clearance of discoloration in our cases adds weightage to this hypothesis. However, lack of properly designed human studies on metabolism of organophosphorus compounds limits any further speculation regarding our hypothesis.

## 4. Conclusion

Blackish discoloration of urine following organophosphorus poisoning is a manifestation that is being increasingly recognized. Though haemolysis caused by certain compounds might account for this discoloration, a class effect due to urinary excretion of metabolites must be considered a more plausible mechanism. Beyond doubt, in cases with blackish discoloration of urine, haemolysis and rhabdomyolysis need to be ruled out. Further studies to elucidate the complex pathways involved in metabolism of organophosphorus compounds are likely to uncover the reasons behind such atypical manifestations and their significance.

## Figures and Tables

**Table 1:** Common clinical features of organophosphorus poisoning based on sites of action [10–12].

Features due to muscarinic receptor overstimulation	Bronchospasm and bronchorrhoea Bradycardia Miosis Diarrhea Salivation Vomiting Lacrimation Excessive sweating

Features due to nicotinic receptor overstimulation in sympathetic system	Mydriasis Hypertension Tachycardia

Features due to nicotinic receptor overstimulation in the CNS	Coma Respiratory failure Confusion

Features due to nicotinic receptor overstimulation at neuromuscular junction	Muscle weakness Fasciculations
